# On the Possibility of Miniature Diamond-Based Magnetometers Using Waveguide Geometries

**DOI:** 10.3390/mi9060276

**Published:** 2018-06-01

**Authors:** Lykourgos Bougas, Alexander Wilzewski, Yannick Dumeige, Dionysios Antypas, Teng Wu, Arne Wickenbrock, Emilie Bourgeois, Milos Nesladek, Hannah Clevenson, Danielle Braje, Dirk Englund, Dmitry Budker

**Affiliations:** 1 Institut für Physik, Johannes Gutenberg-Universität Mainz, 55128 Mainz, Germany; awilzews@students.uni-mainz.de (A.W.); teng@uni-mainz.de (T.W.); wickenbr@uni-mainz.de (A.W.); budker@uni-mainz.de (D.B.); 2 CNRS, UMR 6082 FOTON, Enssat, 6 rue de Kerampont, CS 80518, 22305 Lannion CEDEX, France; yannick.dumeige@univ-rennes1.fr; 3 Helmholtz Institut Mainz, 55099 Mainz, Germany; dantypas@uni-mainz.de; 4 Institute for Materials Research (IMO), Hasselt University, Wetenschapspark 1, B-3590 Diepenbeek, Belgium; emilie.bourgeois@uhasselt.be (E.B.); milos.nesladek@uhasselt.be (M.N.); 5 MIT Lincoln Laboratory, Lexington, MA 02420, USA; hannahclevenson@gmail.com (H.C.); danielle.braje@gmail.com (D.B.); 6 Department of Electrical Engineering and Computer Science, Massachusetts Institute of Technology, Cambridge, MA 02139, USA; englund@mit.edu; 7 Department of Physics, University of California, Berkeley, CA 94720-7300, USA; 8 Nuclear Science Division, Lawrence Berkeley National Laboratory, Berkeley, CA 94720, USA

**Keywords:** diamond-based magnetometer, NV-centers, compact sensor

## Abstract

We propose the use of a diamond waveguide structure to enhance the sensitivity of magnetometers relying on the detection of the spin state of nitrogen-vacancy ensembles in diamond by infrared optical absorption. An optical waveguide structure allows for enhanced optical path-lengths avoiding the use of optical cavities and complicated setups. The presented design for diamond-based magnetometers enables miniaturization while maintaining high sensitivity and forms the basis for magnetic field sensors applicable in biomedical, industrial and space-related applications.

## 1. Introduction

Magnetometers that exploit the spin properties of the negatively charged nitrogen-vacancy (NV) color center in diamond have, in the recent years, achieved exceptional magnetic field sensitivities at room temperature [1,2,3], but these sensitivity levels are still several orders of magnitude above the standard quantum limit [1]. To achieve improved sensitivity limits, diamonds with ensembles of NV centers are implemented, but still, most detection protocols are fluorescence-based, and as such, their magnetometric sensitivities are typically limited by: (a) low conversion efficiency of pump photons into fluorescent photons; (b) background fluorescence; and (c) poor collection efficiency [4]. Recent advances in diamond engineering have allowed for the integration of NV-centers into diamond photonic structures whose optical performance exceeds that offered by bulk diamond crystals and have, thus, enabled improvements in all the aforementioned limiting factors. For example, using fabricated photonic structures directly onto the diamond surface, such as high aspect-ratio tapered pillar-shaped nanowaveguides and/or nanogratings, a 5- to 20-fold enhancement in collection efficiency has been demonstrated [5,6], while alternative approaches make use of microfabricated solid-immersion lenses [7] and optical fibers [8]. Recently, Clevenson and co-workers used a light-trapping diamond waveguide geometry for highly sensitive magnetometric and temperature measurements, that allowed for a combined improvement in excitation efficiency and signal collection of fluorescent photons of more than three orders of magnitude over typical single-pass geometries [9].

**Figure 1:**
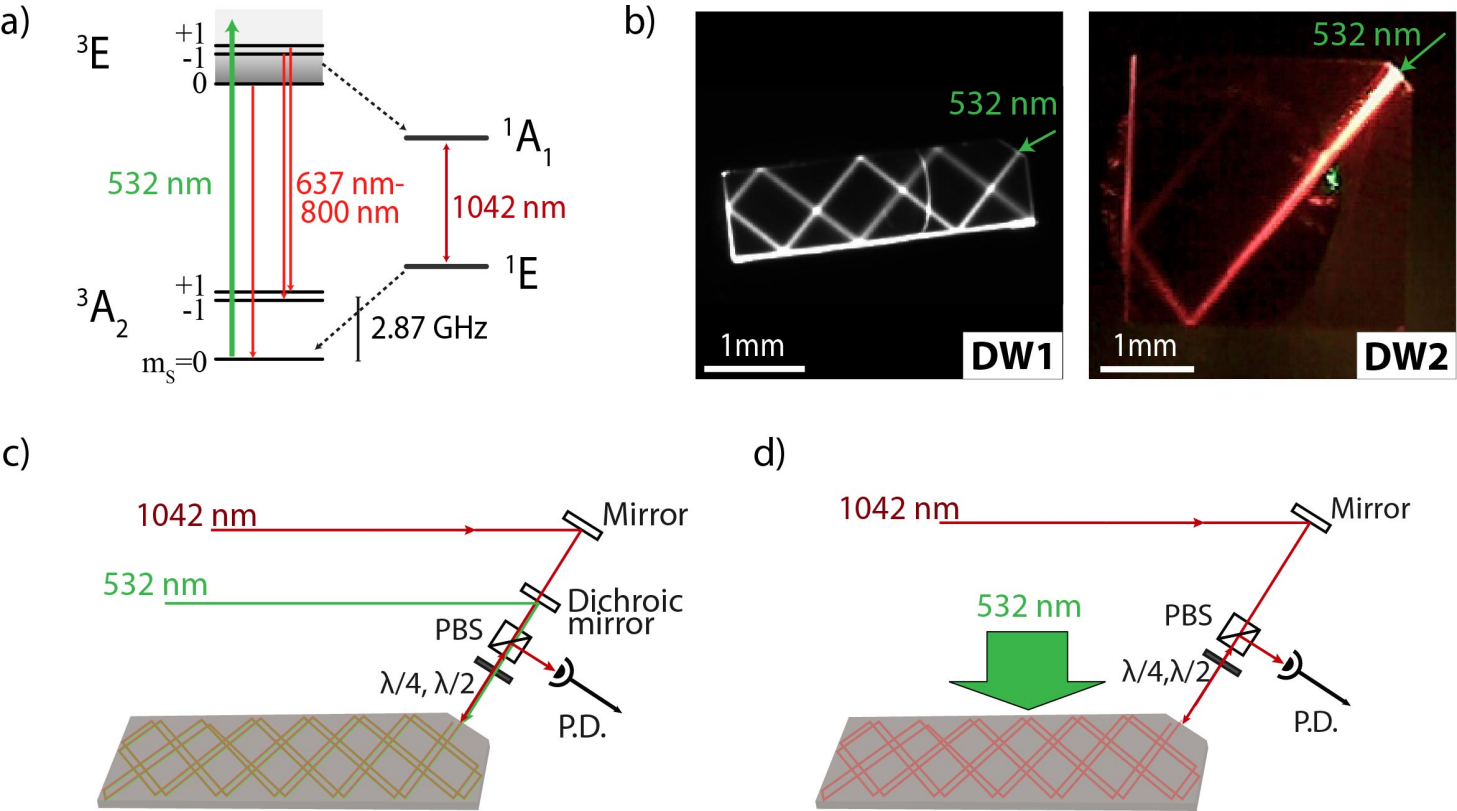
(**a**) Energy-level diagram of the negatively-charged nitrogen-vacancy (NV) color center in diamond. (**b**) Photographs of two different diamond waveguide (DW) crystals used in our experiments, excited by a pump (532 nm) laser (with a power of ≈100 mW) showing bright fluorescence (a long-pass filter with a 600 nm cut-off wavelength is used to record these images). Schematic of infrared (IR)-absorption magnetometric setup for two different configurations: (**c**) the pump beam is overlapped with the IR beam within the waveguide geometry; (**d**) the pump beam is focused through the side of the diamond waveguide allowing for uniform illumination of the waveguide, which ensures maximum spatial overlap between the IR and the pump beam.

An alternative magnetometric technique relies on the detection of the spin state of the NV-center in diamond using the infrared (IR) optical transition at 1042 nm, which is related to the singlet states of the NV-center (see Figure 1a). The 1042 nm transition can be exploited in an IR-absorption scheme to provide higher magnetic-field sensitivities compared to fluorescence-based detection methods [10,11,12]. For an absorption scheme, collection efficiency can approach unity, while measurements are performed in a fluorescence-background-free environment, thereby circumventing the disadvantages of fluorescent-based magnetometric protocols. Chatzidrosos et al. recently presented a miniature cavity-enhanced room-temperature magnetometer based on the IR-absorption protocol with a demonstrated nearly photon shot-noise limited sensitivity of 28 pT/$\sqrt{\mathrm{Hz}}$ and an estimated quantum projection-noise limit of 0.43 pT/$\sqrt{\mathrm{Hz}}$ [13]. We note that additional schemes for performing ensemble magnetometry using the absorption of the pump radiation have also been demonstrated [14].

We present here a new approach towards highly sensitive absorption-based magnetometers using diamond waveguide geometries [9]. The use of a diamond waveguide structure can, for carefully selected geometries, allow for more than ×300 interaction path-length enhancements- compared to single-pass configurations- for the IR probe laser beam, avoiding, thus, the use of resonant optical cavities and complicated optical setups. Moreover, we demonstrate that the proposed sensor can reach sub-pT/$\sqrt{\mathrm{Hz}}$ magnetometric sensitivities. Our design and measurement protocol enable miniaturisation while maintaining high magnetic-field sensitivities and form the basis for magnetic field sensors for biomedical and industrial applications, but also in space-related applications where complexity, size, weight, and the cost of traditional magnetometers have so far prevented their applicability in smaller missions involving, for example, cubesats [15,16].

## 2. Absorption-Based Magnetometry: Principles

Absorption-based magnetometry protocols using NV-centers rely on the ability to optically measure the Zeeman shifts of the NV-center spin sublevels via monitoring of an IR probe beam resonant with the 1042 nm zero-phonon transition between the singlet states of the NV-center (see Figure 1a) [10,11,12]. The NV center is optically excited from the ground state ${}^{3}A_{2}$ to the ${}^{3}E$ state using a laser beam with, typically, a wavelength of 532 nm. From the ${}^{3}E$ state, the NV center can decay to the ${}^{3}A_{2}$ state through a spin-conserving transition leading to fluorescence in the 637–800 nm wavelength range, but it can also decay to the upper singlet state ${}^{1}A_{1}$ through a spin-nonconserving transition which occurs with higher probability for the $m_{s}=\pm 1$ states compared to the $m_{s}=0$ state. From the ${}^{1}A_{1}$ state, the NV center decays to the metastable ${}^{1}E$ singlet state (with a lifetime of ∼200 ns at room temperature) from which it can decay back to the ground state ${}^{3}A_{2}$. Under continuous illumination, the ${}^{1}E$ metastable singlet state is populated and the NV center is mainly pumped to the ${}^{3}A_{2}$ $m_{s}=0$ ground state (Figure 1a).

The absorption of the 1042 nm probe light beam is measured with and without an applied microwave (MW) field resonant to the energy difference between the $m_{s}=0$ and $m_{s}=\pm 1$ spin states, under continuous pumping conditions. For a MW field with frequency (2.87 $\;\pm {\;}^{\gamma }{/}_{2\pi }\cdot B$) GHz, where B is the projection of an externally applied magnetic field along one of the four NV orientations and ${|}^{\gamma }{/}_{2\pi }|≃28$ GHz·T${}^{-1}$ the gyromagnetic ratio of the electron, population is transferred from the $m_{s}=0$ to the $m_{s}=\pm 1$ Zeeman sublevels resulting in greater population in the metastable singlet state, and therefore in higher IR absorption signal. Conversely, without MWs applied, a reduced population in the metastable singlet state results in decreased IR absorption. Considering this, we define the measurement contrast *C* as the relative difference in the IR-absorption signal detected after propagation within a diamond structure:

(1) $$C=\frac{I_{\mathrm{out}}\left(0\right)-I_{\mathrm{out}}\left(\Omega_{R}\right)}{I_{\mathrm{out}}\left(0\right)},$$

where $I_{\mathrm{out}}\left(0\right)$ [$I_{\mathrm{out}}\left(\Omega_{R}\right)$] denotes the IR absorption signal without [with] the application of the MW field whose Rabi frequency is $\Omega_{R}$. The absorption signal $I_{\mathrm{out}}$, is given by the Beer-Lambert law:

(2) $$\frac{I_{\mathrm{out}}}{I_{0}}=e^{-n\cdot \sigma_{\mathrm{IR}}\cdot l},$$

where $I_{0}$ is the input intensity, *n* is the NV density, $\sigma_{\mathrm{IR}}$ is the cross-section for the IR transition ($\sigma_{\mathrm{IR}}=3_{-1}^{+3}\times 10^{-22}$ m${}^{2}$) [11,12], and *l* is the interaction path-length between the IR light and the NV centers. The photon shot-noise limited magnetic field sensitivity for an IR-absorption-based magnetometric protocol is given by [10,11,12]:

(3) $$\begin{array}{ccc} \delta B(T) & = & \frac{\Delta \nu_{\mathrm{mr}}}{\gamma \;C}\;\sqrt{\frac{h\;c}{P_{{}_{\mathrm{IR}}}\;t_{m}\;\lambda_{{}_{\mathrm{IR}}}}}, \end{array}$$

where *h* is the Planck constant, *c* the speed of light, $P_{{}_{\mathrm{IR}}}$ the measured IR probe beam signal output power ($\lambda_{{}_{\mathrm{IR}}}=1042$ nm), $t_{m}$ is the measurement time, and $\Delta \nu_{\mathrm{mr}}$ is the full-width-at-half-maximum (FWHM) linewidth of the observed magnetic spin-resonance. The magnetic spin-resonance linewidth $\Delta \nu_{\mathrm{mr}}$ is fundamentally limited by the dephasing rate T${}_{2}^{*}$ of the NV-center electron spin [17].

Equation (3) reveals that the photon shot-noise magnetic-field sensitivity is mainly limited by the contrast *C* and the rate of detected IR photons. Therefore, in an absorption-based magnetometric protocol, one needs to maximise the measurement contrast, which is proportional to the total absorption of the IR probe beam by the NV centres, while ensuring sufficient IR photon collection rates.

**Figure 2:**
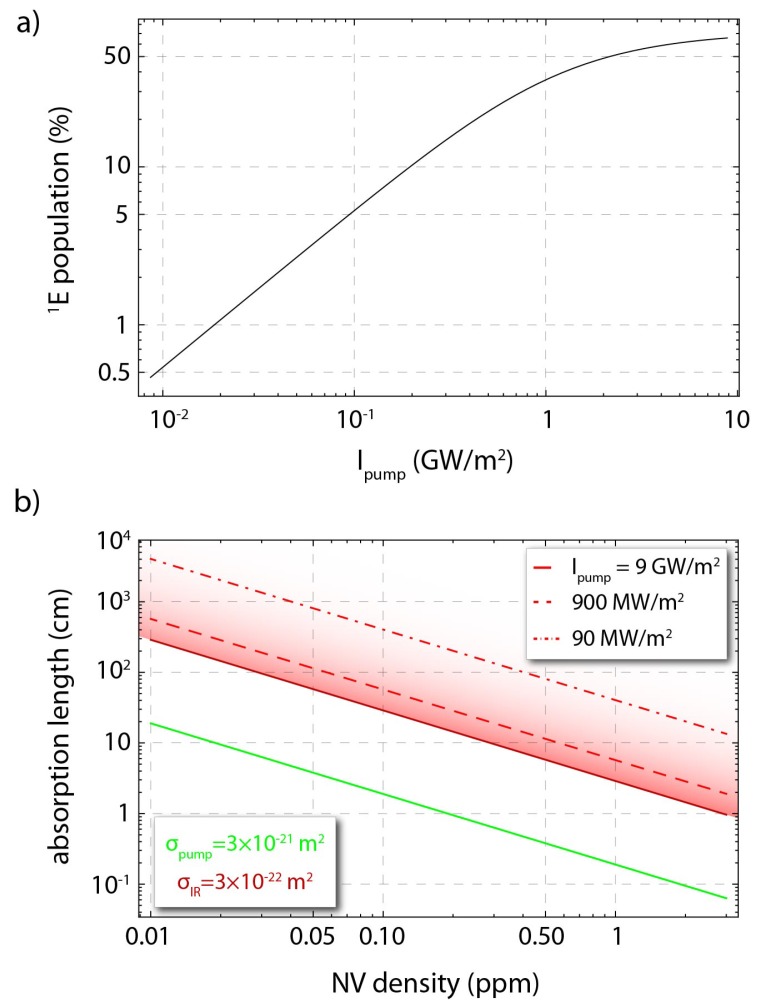
(**a**) Estimated population in the singlet ${}^{1}$E state as a function of the pump (532 nm) intensity. (**b**) Absorption lengths as a function of the NV-density, for the pump radiation (green solid, line), and for the IR (1042 nm) probe radiation (red solid, dashed, and dotted, lines) for three different pump intensities (which yield different populations in the ${}^{1}$E state). Simulations are based on a rate-equation model for the NV center following the works in Refs. [11,18].

The cross-section for the IR transition is approximately an order of magnitude smaller than the cross section for the pump transition at 532 nm [$\sigma_{532\;\mathrm{nm}}=3\left(1\right)\times 10^{-21}$ m${}^{2}$] [11,12]. Hence, in order to obtain a significant absorption signal and, thus, a large contrast, one needs, for a given NV density, to maximise the interaction path-length between the IR light and the NV centers. We use a rate-equation model for the NV-center to estimate the absorption length for the IR transition. We follow the model presented in Ref. [11], and we also consider the estimations for ionization and recombination rates as presented in Ref. [18]. Figure 2a presents the population percentage in the ${}^{1}$E singlet state as a function of the pump intensity, and Figure 2b the absorption lengths for the pump and IR probe beams as a function of the NV-density and the pump intensity. We see that for moderate NV-densities (∼0.1–1 ppm), for which we can expect small differences in the coherence properties of the NV centers due to interactions with their environment [1], IR absorption lengths of 3–30 cm are expected, and thus, enhancements that yield similar path-lengths are necessary for sensitive absorption-based magnetometry.

Thus far, cavity-enhanced schemes have been employed for enhancing the IR interaction/absorption path-length, with demonstrated enhancements of ∼×100 and attainable effective interaction path-lengths of a few centimetres [12,13]. While these cavity-enhanced schemes have resulted in shot-noise limited magnetometric sensitivities, they still suffer from disadvantages such as: (a) the requirement for optics that form an optical cavity, (b) the requirement for especially designed low-loss optical coatings on the diamond crystal surfaces, and (c) the need for electronics that maintain a stable frequency-lock between the IR laser frequency and the optical cavity.

## 3. Diamond Waveguides

We consider a diamond waveguide consisting of a rectangular cuboid diamond crystal with a small angled facet at one corner for input and output coupling of a laser beam. This design can allow for enhanced IR-absorption path-lengths while avoiding the drawbacks of cavity-enhanced schemes. In particular, for an absorption-based magnetometry protocol, IR laser light is coupled and trapped within the diamond waveguide due to total internal reflections. After several reflections within the waveguide, IR light exits through the same angled facet and its absorption is monitored in the presence of optical and MW excitation of the NV centers.

Figure 1b shows images of two diamond waveguides (DW) we are using in our experiments (hereafter referred to as DW1 and DW2).

DW1 consists of a 〈100〉-oriented, type IIa, diamond grown by chemical vapour deposition (CVD) with dimensions 3mm×1mm×300 μm (Figure 1b), and is prepared following a procedure similar to the one described in Ref. [9]. Using the measured absorption of green light through DW1, we estimate an absorption length at 532 nm of ∼1 cm, which corresponds to an NV-density for DW1 of approximately 0.2(1) ppm.

DW2 is based on a diamond crystal synthesised at the Hasselt University using a home-made microwave plasma CVD reactor. As a growth substrate we use a commercially acquired, 〈100〉-oriented, CVD diamond plate, and the growth conditions for DW2 are similar to the ones described in Ref. [19]. DW2 has dimensions of 3mm×3mm×300 μm (Figure 1b). The sample was electron-irradiated at 14 MeV with an irradiation dosage of 8 $\times \;10^{18}$ e${}^{-}$/cm${}^{2}$, and annealed for 2 h at 1050 ${}^{\circ }$C. Using the measured absorption of green light through DW2, we estimate an absorption length at 532 nm of ∼1.4 mm (Figure 1b), which corresponds to an NV-density for DW2 of approximately 1.3(1) ppm. We note that for both waveguides, we perform absorption measurements over different areas to verify that the density of NV-centres is uniform.

**Figure 3:**
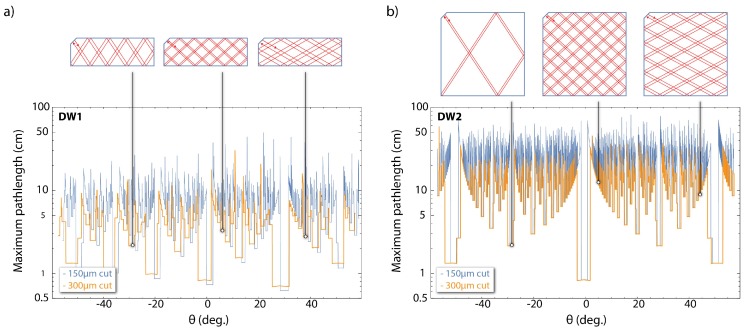
Numerical simulations of the maximum optical path-lengths of the IR probe radiation plotted as a function of the input angle and for two different input/output facet-cut lengths (300 μm and 150 μm) for the two different waveguide geometries we use: (**a**) DW1 and (**b**) DW2. Facet cuts are at a 45 deg. angle with respect to the diamond crystal edges. The IR laser beam intersects the input facet 50 μm from the centre. We also show examples of the predicted IR optical path-lengths within the different waveguide geometries for different input angles.

In Figure 3 we present numerical simulations for the maximum attainable IR optical path-lengths as a function of the IR beam incidence angle. For these simulations (done for both DW1 and DW2 geometries) we consider an ideal probe beam (infinitely thin with infinite Rayleigh range), and a refractive index of diamond of η=2.391 for 1042 nm [20], yielding a critical angle of $\theta_{c}=24.72^{\circ }$. In addition, using different input/output facet length-cuts we demonstrate how the maximum optical path-length depends on the length cuts. Furthermore, we observe for the DW1 waveguide geometry possible optical absorption path-lengths of ∼30 cm, resulting in path-length enhancements of >100 compared to a single-pass configuration, while for DW2, optical absorption path-lengths of ∼60 cm, resulting in path-length enhancements of >200. These predicted path-lengths are almost an order of magnitude larger than the attainable absorption path-lengths using cavity-enhanced schemes [12,13]. We emphasise that, to achieve these optical path-lengths on a device employing a waveguide structure, and ensure that IR light eventually exits the device and is recorded, one needs a diamond crystal that is as close to a rectangular cuboid as possible and has high surface quality and optical flatness.

## 4. Absorption-Based Magnetometry Using a Diamond Waveguide

### 4.1. Preliminary Experimental Results

As described previously, for highly sensitive absorption-based magnetometry one must achieve significantly high populations in the ${}^{1}$E state, which requires strong pump intensities (see Figure 2a), and large IR optical path-lengths. For the waveguides we use in our experiments, we expect large IR optical path-lengths (>10 cm; Figure 3). In Figure 2b we show the absorption length for the pump radiation as a function of the NV density. For an [NV] ∼ 0.2 ppm the absorption length for 532 nm is estimated to be ∼0.94 cm, while for an [NV] ∼ 1 ppm this length is estimated to be ∼0.19 cm.

To achieve high IR absorptions we use two alternative experimental configurations as shown in Figure 1c,d. In particular, to ensure high pump intensities for low-density diamond samples (DW1 in our case), and achieve the desired high IR absorption, we use a configuration in which the pump and IR beams are overlapped (Figure 1c). This configuration becomes inadequate for long optical path-lengths (>3 cm) with DW1 due to pump depletion, and for the high-density diamond sample (DW2) in which the absorption length for the pump beam is comparable to the width of the diamond crystal. For these reasons, we use a side-pumping configuration [Figure 1d] which enables us to avoid pump depletion, while ensuring that NV-centres are pumped uniformly.

In our experiments, the pump radiation is provided by a diode-pumped solid-state laser (gem532 laser, 2 W max. power; LaserQuantum, Stockport, UK), while the IR radiation is provided by an external-cavity diode laser (DL-Pro, 100 mW max. power; Toptica, Graefelfing, Germany). In the overlapped configuration, a dichroic mirror is used to combine the pump and IR beam. In the side-pumping configuration we use an anamorphic prism pair to shape one dimension of the laser pump beam such that it matches the length of the waveguide, and a cylindrical lens to tightly focus the other dimension of the pump beam within the waveguide. We use a camera to image the pump and IR optical path-lengths within the diamond waveguides (CMOS camera DMK23UP031, Bremen, Germany) (camera is not depicted in Figure 1c,d), whose input is spectrally filtered with the use of appropriate optical filters (NF533-17 (Thorlabs Inc., Newton, NJ, USA) green notch filter for blocking scattering from the pump beam; FELH0600 (Thorlabs Inc., Newton, NJ, USA) for imaging the NV fluorescence; FEL0950 (Thorlabs Inc., Newton, NJ, USA) for imaging the IR beam). Finally, for both experimental configurations, we simultaneously monitor fluorescence and IR-absorption, and optically detect magnetic resonances (ODMR) of the NV-centers in both of these signals.

**Figure 4:**
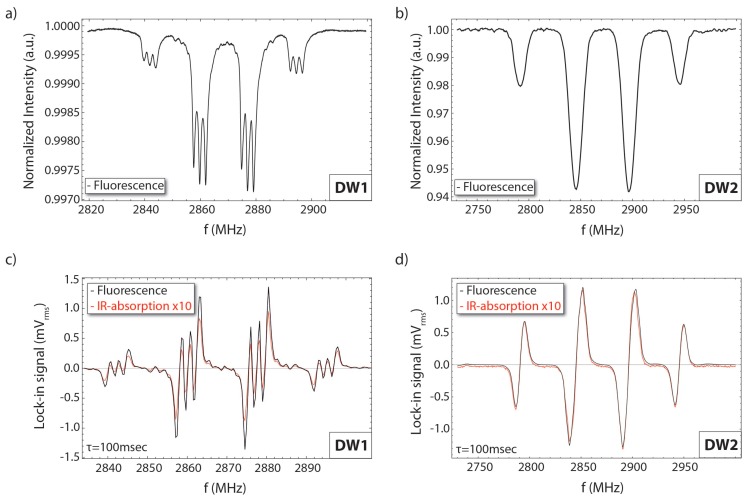
Continuous-wave optically detected magnetic resonance (ODMR) spectra obtained from the emitted fluorescence from (**a**) DW1, and (**b**) DW2. Frequency-modulation spectroscopy of ODMR spectra in fluorescence (black lines) and absorption (red lines) for (**c**) DW1 and (**d**) DW2. See text for further details. All presented data were obtained with an external applied magnetic field aligned along the diamond’s [111] crystal-axis under continuous pumping and MW excitation.

In Figure 4 we present ODMR spectra obtained in the fluorescence and absorption channel using both diamond waveguides (DW1 & DW2), under continuous-wave pumping and MW field excitation. The MW field is provided by a 200 μm-diameter wire loop placed above the diamond waveguide. We use an aspheric lens to collect the fluorescence from the side of each waveguide structure, which is then focused on a photodiode (Thorlabs PDA100A2). A polarising beam splitter and an appropriate waveplate allows for the collection of the backward-directing IR probe light exiting the waveguides, which is then focused on a photodiode (Thorlabs PDA36A2). For all measurements shown in Figure 4, we apply a constant background magnetic field of ∼10–20 G along the [111] diamond crystal axis using permanent magnets. The externally applied magnetic field lifts the degeneracy of the NV spin sub-level transitions $m_{s}=0\to \pm 1$. We observe that both DW1 & DW2 have NV centers in all four possible orientations supported by the crystal lattice.

For the fluorescent measurements using DW1 (Figure 4a), we use low pump and MW power levels and we observe ODMR resonances that have a FWHM linewidth of approximately ∼1 MHz. Under these conditions, the ODMR linewidth is limited by the T${}_{2}^{*}$ coherence of the diamond, which is found to be ∼320 ns. Moreover, in DW1 we clearly observe the hyperfine coupling of the NV-centers to the ${}^{14}$N nuclear spin (*I* = 1), which splits each of the spin sub-level transitions into a triplet. For the fluorescence measurements done with DW2 (Figure 4b), we use a side-pumping configuration, in which the pump and MW power levels are such that the ODMR features are power broadened. Independent measurements, however, have allowed us to identify the T${}_{2}^{*}$ coherence time to be ∼100 ns.

Figure 4c,d present measurements of frequency-modulation (FM)-spectroscopy of the ODMR absorption signals using DW1 and DW2, respectively, together with the corresponding fluorescence signals for comparison. For the data taken with DW1 (DW2), the modulation frequency is chosen to be 11.2 kHz with a corresponding modulation depth of ∼1 MHz (5 MHz). To acquire data we use a lock-in amplifier (SR7265; Signal Recovery), with a measurement time constant of 100 ms. Each data point in Figure 4c,d is the average of 50 measurements made for the particular frequency.

For the results shown in Figure 4c,d, the IR optical absorption-path-length realised using DW1 (DW2) is ∼1.5 cm (∼2.8 cm). In the case of DW1, where we use overlapped pump-IR beams, we have been unable to directly image the IR optical path-length within the waveguide but we are able to infer it from the pump optical path-length (in particular, bright IR scattering-background from the waveguide edges prohibits sensitive imaging of the IR optical path within the diamond). In the case of DW2, where we use side-pumping due to the high NV-density, we are able to image the IR path-length within the diamond waveguide through visible path-related bulk scattering.

The results shown in Figure 4c correspond to an IR absorption of ∼0.18% (Equation (2)) (DW1; overlapped green and IR beams, optical path-length of 1.5 cm), and the results shown in Figure 4d to an IR absorption of ∼0.3% (DW2; side-pumping configuration, optical path-length of 2.8 cm). Using DW1 and overlapped pump and IR beams, we are able to achieve relatively high pump intensities and significant population in the ${}^{1}$E state, but the low NV density effectively results in low absorption levels. Conversely, for the measurements with DW2 we obtain relatively low absorption levels, as a consequence of the low pump intensities we are able to achieve in our side-pumping setup. Furthermore, the presented results (Figure 4c,d) correspond to magnetometric sensitivities of approximately ∼100–600 nT/$\sqrt{\mathrm{Hz}}$. Our main current limitation in obtaining better magnetometric sensitivities is associated with large losses in IR power for larger optical absorption path-lengths. These losses limit us to low IR photon collection rates and to low absorption signals and, consequently, ODMR contrasts. In particular, for both waveguide geometries and path-lengths greater than ∼3 cm, the collected IR light drops to lower than <10 μW (for an incident IR power of ∼30 mW). We have identified that this issue is related to reflection losses from the waveguide surfaces, as well as from large losses from reflections on the edges and corners of the waveguides (in the case of DW2 we have additional bulk scattering losses). For this reason, we have been unable to achieve larger path-lengths and obtain higher IR absorption levels (for comparison, in Ref. [13] absorption levels of ∼50% near saturation were possible for an effective IR absorption-path-length of ∼5 cm and a diamond crystal with [NV] ∼ 2 ppm). For future implementations we plan for higher quality polishing of our waveguides, that will result in lower surface roughness, thus improving the TIR within the waveguides and reducing scattering losses. We note that more complicated diamond geometries (e.g., a polyhedron, cube) tailored specifically for maximum optical path-lengths can be more appropriate for practical implementation of our presented magnetometric technique.

### 4.2. Discussion and Projected Sensitivities

**Figure 5:**
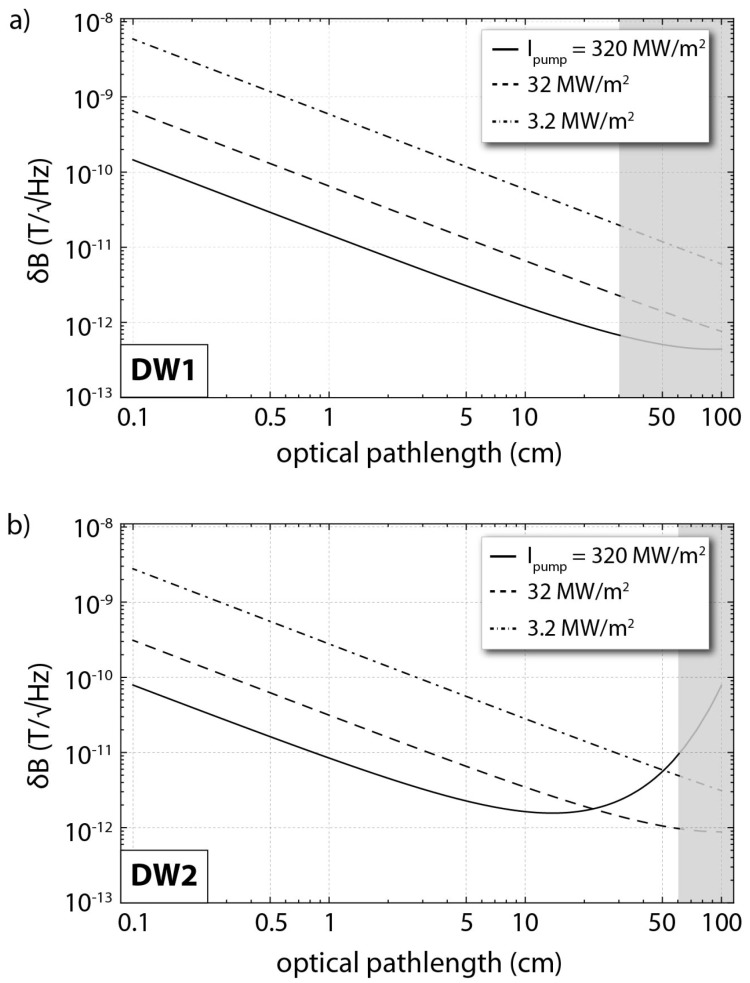
Projected IR photon shot-noise limited magnetic-field sensitivities as a function of the IR optical absorption path-length for and absorption-based magnetometric protocol employing (**a**) DW1, and (**b**) DW2. For the simulations we assume an input IR power of 30 mW and a MW field with Rabi frequency of $\Omega_{R}=2\pi \times 1$ MHz. The shaded areas correspond to path-lengths not realisable with the corresponding waveguide, following the simulations shown in Figure 3.

From our preliminary measurements we can estimate the photon shot-noise limited magnetic-field sensitivity of an absorption-based magnetometric protocol employing diamond waveguides similar to the ones presented here. In particular, in Figure 5 we present simulations for the IR photon shot-noise limited magnetic-field sensitivities using DW1 and DW2. Assuming a configuration resulting in an IR optical path-length of 10 cm within DW2 and a pump intensity of ∼320 MW/m${}^{2}$, we estimate an absorption of ∼67%, and for MW fields with Rabi frequency of $\Omega_{R}=2\pi \times 1$ MHz, an IR photon shot-noise magnetic field sensitivity of ∼1.6 pT/$\sqrt{\mathrm{Hz}}$. For higher optical path-lengths and strong pump intensities, the collected photon rate becomes significantly smaller due to high absorption, resulting in poor magnetic field sensitivities. We also predict sub-pT/$\sqrt{\mathrm{Hz}}$ sensitivities for optical path-lengths of ≳30 cm using either DW1 or DW2 (see Figure 5 for large optical path-lengths). We note again that in our current setups we are not able to reach the estimated magnetic field sensitivities as we are limited in pump intensity (maximum ∼5 MW/m${}^{2}$ for the side-pumping configuration) and in the effective IR collected power when working at large optical path-lengths (P${}_{\mathrm{IR}}<$ 10μW).

An additional critical parameter is the capability to obtain uniform and strong MW fields over the whole area of the waveguides. Different MW-system designs have been proposed for achieving uniform and large-area MW coupling to NV centers [21,22], but these are limited to areas of ∼1 mm${}^{2}$ and are, therefore, inadequate for efficient MW-coupling in complex IR path-lengths similar to the ones depicted in Figure 3. There exist several MW resonators designed for electron paramagnetic resonance (EPR) experiments, which are based on standing-wave cavities and are optimised for large samples. For these resonators, B${}_{1}$ field strengths of the order of a few Gauss are expected and can be uniform over areas similar to the areas of DW1 and DW2 [23,24], making them ideal for implementation with our system. An alternative solution is to employ MW-free magnetometric protocols which are based on the properties of the NV-center ground-state level anti-crossings [25,26].

In conclusion, we have presented here an absorption-based magnetometer consisting of diamond waveguides that allow for absorption path-length enhancements of >300. Our sensor does not require use of complicated setups and electronics, and has a projected shot-noise magnetometric sensitivitiy of sub-pT/$\sqrt{\mathrm{Hz}}$. These characteristics and its potential combination with MW-free protocols, makes this device a potential candidate for space-related applications in which mass, volume and power savings are important issues [27].

## Abbreviations

The following abbreviations are used in this manuscript:

NV	Nitrogen Vacancy
MW	Microwave
FWHM	Full Width Half Maximum
ODMR	Optically Detected Magnetic Resonance
DW1	Diamond Waveguide 1
DW2	Diamond Waveguide 2
